# Linezolid Pharmacokinetic-Anemia Modeling in Children With Rifampicin-Resistant Tuberculosis

**DOI:** 10.1093/cid/ciae497

**Published:** 2024-10-18

**Authors:** Jordan T Brooks, Belén P Solans, Agathe Béranger, H Simon Schaaf, Louvina van der Laan, Sangeeta Sharma, Jennifer Furin, Heather R Draper, Anneke C Hesseling, Anthony J Garcia-Prats, Radojka M Savic

**Affiliations:** University of California–San Francisco, Department of Bioengineering and Therapeutics, School of Pharmacy, San Francisco, California, USA; University of California–San Francisco, Department of Bioengineering and Therapeutics, Schools of Pharmacy and Medicine, San Francisco, California, USA; UCSF Center for Tuberculosis, University of California–San Francisco, San Francisco, California, USA; University of California–San Francisco, Department of Bioengineering and Therapeutics, Schools of Pharmacy and Medicine, San Francisco, California, USA; UCSF Center for Tuberculosis, University of California–San Francisco, San Francisco, California, USA; URP7323 Pharmacologie et Évaluation des Thérapeutiques Chez l'Enfant et la Femme Enceinte, 21 Université Paris Cité, Paris, France; Desmond Tutu TB Centre, Department of Paediatrics and Child Health, Faculty of Medicine and Health Sciences, Stellenbosch University, Cape Town, South Africa; Desmond Tutu TB Centre, Department of Paediatrics and Child Health, Faculty of Medicine and Health Sciences, Stellenbosch University, Cape Town, South Africa; Division of Clinical Pharmacology, Department of Medicine, University of Cape Town, Cape Town, South Africa; Department of Pediatrics, National Institute of Tuberculosis and Respiratory Diseases, New Delhi, India; Department of Global Health and Social Medicine, Harvard Medical School, Boston, Massachusetts, USA; Desmond Tutu TB Centre, Department of Paediatrics and Child Health, Faculty of Medicine and Health Sciences, Stellenbosch University, Cape Town, South Africa; Desmond Tutu TB Centre, Department of Paediatrics and Child Health, Faculty of Medicine and Health Sciences, Stellenbosch University, Cape Town, South Africa; Desmond Tutu TB Centre, Department of Paediatrics and Child Health, Faculty of Medicine and Health Sciences, Stellenbosch University, Cape Town, South Africa; Department of Pediatrics, School of Medicine and Public Health, University of Wisconsin–Madison, Madison, Wisconsin, USA; University of California–San Francisco, Department of Bioengineering and Therapeutics, Schools of Pharmacy and Medicine, San Francisco, California, USA; UCSF Center for Tuberculosis, University of California–San Francisco, San Francisco, California, USA

**Keywords:** linezolid, anemia, children, dosing, pharmacokinetics

## Abstract

**Background:**

Linezolid, a component of rifampicin-resistant/multidrug-resistant tuberculosis (RR/MDR-TB) treatment, is associated with treatment-limiting toxicities, including anemia. Patient-level and linezolid pharmacokinetic risk factors for anemia have not been well described in children treated for RR/MDR-TB.

**Methods:**

We evaluated the pharmacokinetics of linezolid and longitudinal hemoglobin data to validate an existing population linezolid pharmacokinetic model. We assessed the impact of linezolid pharmacokinetics and the risk of developing anemia in a prospectively enrolled cohort of children. A previously published population pharmacokinetic linezolid model was validated using nonlinear mixed effects modeling. A multivariable ordinal logistic regression model was built to predict the incidence of anemia.

**Results:**

A total of 112 children, median age 7.2 years (interquartile range, 2.2–16.3), were included from South Africa (n = 87) and India (n = 25). Of these, 24 children contributed new linezolid pharmacokinetic data. The population pharmacokinetic model, which informs the currently recommended linezolid dosing in children (10–15 mg/kg), was validated with these additional new data. For every 1 g/dL lower baseline hemoglobin level, the odds of developing grade 3 or 4 anemia increased by 2.64 (95% confidence interval [CI], 1.98–3.62). For every 1 mg/L × h higher linezolid area under the concentration-time curve, the odds of developing grade 3 or 4 anemia increased by 1.012 (95% CI, 1.007–1.017).

**Conclusions:**

Taken together, these data confirm currently recommended linezolid doses for children. The risk of anemia in children should be carefully considered and monitored. Initiating linezolid in children with low baseline hemoglobin increases the probability of experiencing grade 3 or 4 anemia.

## Teaser

We used previously developed population pharmacokinetic model–based predictions to analyze new linezolid pharmacokinetic data to confirm linezolid dosing in children. At currently recommended doses, children with low baseline hemoglobin or high linezolid exposure are at higher risk of anemia. An existing population pharmacokinetic model was used to describe new, prospectively collected linezolid pharmacokinetic data in children with rifampicin-resistant tuberculosis (RR-TB). The model predicted the data well. No adjustments to current linezolid dosing recommendations are required. Low baseline hemoglobin and higher linezolid exposures predicted anemia risk in children. These are important baseline markers for potential toxicity in children who require linezolid for RR-TB treatment.

The treatment of rifampicin-resistant/multidrug-resistant tuberculosis (RR/MDR-TB; ie, disease caused by *Mycobacterium tuberculosis* strains resistant to rifampicin [RR] or to both isoniazid and rifampicin [MDR]) remains a significant problem globally. Linezolid is commonly used in RR/MDR-TB treatment regimens; it is an oxazolidinone that inhibits bacterial protein synthesis [1]. As informed by its favorable efficacy in several systematic reviews, linezolid is classified as a priority (group A) drug for use in RR/MDR-TB treatment regimens for children, adolescents, and adults by the World Health Organization (WHO), given its improved TB treatment outcome [2–4].

Linezolid is associated with dose- and duration-dependent adverse effects including myelosuppression (anemia, neutropenia, thrombocytopenia), peripheral neuropathy, optic neuritis, and lactic acidosis [5]. Historically, linezolid has primarily been used for short-course (<28 days) treatment of gram-positive bacterial infections; for these indications, severe adverse effects are relatively infrequent [5]. However, with the long duration of treatment for RR/MDR-TB, typically several months, linezolid-related adverse effects have been reported in up to 60% of people treated long term for RR/MDR-TB, with doses >600 mg in adults being a risk factor for adverse effects [2, 3, 6, 7].

Given the considerable burden of MDR-TB among children and adolescents globally, with 25 000–32 000 new cases annually, and the increased use of linezolid in RR/MDR-TB treatment regimens, we evaluated the pharmacokinetics and safety of linezolid in children routinely treated for RR/MDR-TB [8–10]. A population pharmacokinetic model of linezolid was developed and used to inform weight-based dosing bands by formulation to ensure that children achieve linezolid exposure that is comparable to exposure for adults, that is, the currently recommended 600 mg once daily linezolid dose (area under the time-concentration curve over 24 hours at steady state [AUC_0–24_], 107 mg/L × h) [9, 11]. In this cohort, 10 of 17 (59%) children who received long-term linezolid experienced adverse effects; 5 of 17 (29%) experienced grade 3 or 4 anemia, resulting in discontinuation of linezolid in 4 children. Linezolid-related adverse effects were strongly associated with exposures above the adult target exposures, which resulted in lower proposed weight-based dosing [7, 9].

Our aim in this study was to evaluate the previously described population pharmacokinetic model for linezolid in children and evaluate patient-level and pharmacokinetic drivers of anemia in a larger cohort of prospectively enrolled children and adolescents with RR/MDR-TB [9].

## METHODS

### Study Population

Data for this study came from 4 cohorts of children (aged 0–17 years) routinely treated for RR/MDR-TB in South Africa and India. Most data came from 2 prospective observational pharmacokinetic studies in Cape Town, South Africa. The first (MDRPK1) and second (MDRPK2) studies included children who routinely received RR/MDR-TB treatment that included linezolid. Additionally, in the MDRPK2 study, children who were not routinely treated with linezolid were given a single dose of linezolid on the day of pharmacokinetic sampling to provide additional data for the evaluation of linezolid pharmacokinetics. This subgroup was not included in the long-term safety analysis. Data from the MDRPK1 and MDRPK2 studies were previously used in 2019 to develop a population pharmacokinetic model for linezolid; these data were used to develop the current WHO-recommended weight-based dosing for linezolid [7, 9, 11]. Subsequent pharmacokinetic data were collected as part of the MDRPK2 study after the original population pharmacokinetic model was published and were included in the current analysis.

Additional data were supplied from South African and Indian children who received RR/MDR-TB treatment that included linezolid. Linezolid pharmacokinetic data were not collected, but detailed linezolid dosing and safety data were available. RR/MDR-TB treatment in all children followed national and international guidelines and included a minimum of 4 medications with likely or confirmed susceptibility for a treatment duration of 9–18 months. Clinical decisions regarding the choice of regimen, duration of treatment, and doses used were individualized at the discretion of routine treating clinicians. The linezolid dosing recommendation, which follows the 2019 initial analysis, was 10–15 mg/kg/d [5].

### Population Pharmacokinetic Model and Dosing Simulations

Validation and refinement of the previously published population pharmacokinetic model involved a population nonlinear mixed-effects modeling approach that used NONMEM v.7.5 (ICON Development Solutions, Ellicott City, MD), using the first-order conditional estimation with interaction method. For validation, the model fit of new prospectively collected linezolid pharmacokinetic data was assessed using goodness-of-fit plots and visual predictive checks (VPCs). The new pharmacokinetic data were combined with the original dataset to create an expanded dataset to re-estimate the model parameters and explore refinement of the model if it did not fit well. Linezolid concentrations below the lower limit of quantification (0.100 μg/mL) were excluded from analysis. The covariates that were originally explored but not included in the final model and between-occasion variability were reevaluated during model refinement. Model evaluation was done using goodness-of-fit plots and VPCs. R software (version 3.5.1) with the xpose package (version 4.6.1) [12] was used for dataset review and graphical evaluation of results. After the final model parameters were re-estimated, the linezolid AUC_0–24_ was obtained using the current dosing algorithm to determine whether any adjustments were necessary based on the additional pharmacokinetic data.

### Safety Analyses

Anemia was graded according to Division of AIDS criteria [13] and assessed for attribution to linezolid based on the investigator’s judgment. In the MDRPK1 and MDRPK2 studies, children had regular standard clinical and laboratory safety assessments, including a monthly complete blood count for the first 6 months, then every 2 months or as clinically indicated until completion of treatment. For children from India, hemoglobin data were collected at baseline, monthly for the first 6 months, every 3 months through the first year of treatment, and then at months 18 and 24 for those still being treated at that time. Management of linezolid-associated adverse effects depended on the event type and severity and the clinical context, based on clinician judgment. Lower-grade anemia events (grades 1 or 2) typically resulted in temporary interruption of linezolid treatment, followed by restarting at a reduced dose; higher-grade events (grades 3 or 4) typically resulted in permanent discontinuation, depending on available treatment options and disease severity. For this study, participants who received 1 dose of linezolid for pharmacokinetic analysis and those who were receiving RR/MDR-TB treatment that did not include linezolid were included and categorized as having received no long-term linezolid. Given that anemia can occur due to multiple factors independent of linezolid treatment, the “no long-term linezolid” group provided a meaningful comparator to quantify the increased potential risk of linezolid-related anemia for children with long-term linezolid exposure.

Baseline characteristics were presented using descriptive statistics. Weight-for-age *z* (WAZ) scores were calculated using British reference values, as WHO references include only children aged <10 years [14]. Baseline hemoglobin was defined as hemoglobin measured at the start of enrollment and/or RR/MDR-TB treatment. Because the children enrolled in the MDRPK1 and MDRPK2 studies could have received up to 12 weeks of RR-TB treatment prior to enrollment, some individuals did not have baseline hemoglobin prior to linezolid treatment available for this analysis. Additionally, if treatment with linezolid was temporarily interrupted and then restarted, new baseline hemoglobin was not assigned, and anemia events after linezolid discontinuation were not evaluated. With linezolid dose reduction, the new AUC_0–24_ was then used to evaluate linezolid exposure as a predictor of anemia following linezolid dose reduction. We considered each measured hemoglobin value and corresponding anemia event classification as distinct events. A multivariable ordinal logistic regression model was developed in a stepwise fashion to predict the graded anemia events. In development of the multivariable ordinal regression model, age, sex, linezolid treatment duration, cumulative exposure, baseline hemoglobin, study site, dose received above the current linezolid recommendation, linezolid AUC_0–24_, time, and WAZ scores were explored. The ordinal logistic regression model fit was evaluated using the Akaike information criteria to determine the inclusion of independent variables. Additionally, the significance of multiple included variables was assessed using the Wald test; variables that did not reach a *P-*value threshold of .05 were not included in the final model.

### Ethical Considerations

All studies were approved by local ethics review committees. This pooled analysis was also approved by the Stellenbosch University Health Research Ethics Committee. Written informed consent was obtained from parents/legal guardians on behalf of their children, and written informed assent was obtained from children aged >7 years.

## RESULTS

### Study Population and Pharmacokinetic Model Validation and Refinement

**Figure 1.**
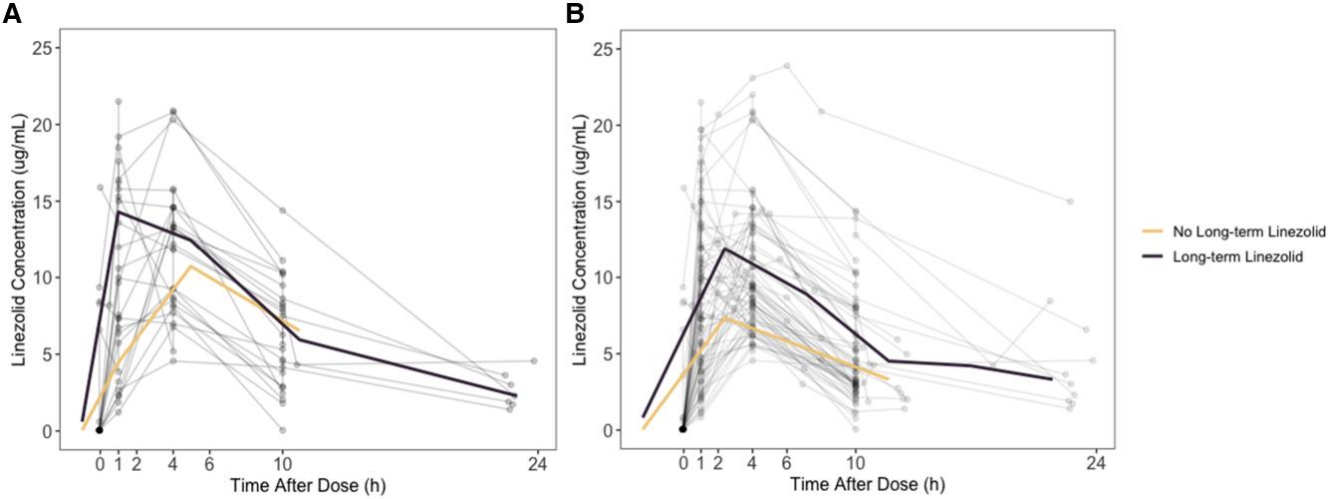
Linezolid concentrations over time in new pharmacokinetic data collected for data validation (*A*) and combined pharmacokinetic data used in model refinement (*B*).

A total of 112 children were included in the safety analysis; 72 children, 48 from the original study and 24 children who provided the new pharmacokinetic data, informed the current model. Participants’ demographic characteristics are summarized in Table 1. The 24 children who provided the new pharmacokinetic data contributed 63 new linezolid samples (Supplementary Table 1). Three (4.8%) pre-dose linezolid concentrations were below the limit of quantification and were not included. One linezolid concentration that suggested an increase in concentration over time without an additional linezolid dose was also removed from analysis. The concentration-over-time profiles of the new pharmacokinetic data and the complete dataset are shown in Figure 1. Both the reevaluation of patient covariates and the inclusion of between-occasion variability did not lead to significant improvement in the model fit. Age was explored in linear, logistic, and logarithmic relationships with clearance of linezolid and was not included in the final model.

**Table 1.** Demographic and Clinical Data of Child Participants of the Linezolid Pharmacokinetic and Safety Studies Included in the Current Study

Characteristic	South Africa	India	MDRPK1 and MDRPK2 Studies, South Africa	Total
Participants	3	25	84	112
Linezolid exposure groups, n				
No long-term linezolid				
None	0	0	12	12
Single dose	0	0	40	40
Long-term linezolid				
Multiple dose	3	25	32	60
Linezolid concentration, n				
Participants with concentration data, n	0	0	72	72
Total concentration samples, n	0	0	329	329
Below the limit of quantification, n (%)	0	0	46 (14)	46 (14)
Concentration, median (range) in mg/L	0	0	7.5 (0.6–28.8)	7.5 (0.6–28.8)
Duration on linezolid, median (range), m	3.1 (3.1–6.4)	24 (0.1 -25.6)	18 (3.1–22.2)	18.2 (0.1–25.6)
Baseline hemoglobin, median (interquartile range), g/dL	12.2 (11.7–12.7)	10.7 (9.2–12.0)	11.6 (10.8–12.7)	11.6 (10.4–12.6)
Sex, n (%)				
Female	2 (66.6)	18 (72.0)	44 (52.4)	64 (57.1)
Male	1 (33.3)	7 (28.0)	40 (47.6)	48 (43.9)
Human immunodeficiency virus–positive, n (%)	0	0	2 (2.4)	2 (1.8)
Age, median (range), y	6.7 (2.1–9.6)	14.0 (8.0–15)	4.3 (0.3–16.3)	7.2 (0.3–16.3)
Weight, median (range), kg	19.0 (13.0–25.0)	28 (13–56)	13.0 (6.3–57.6)	17.8 (6.3–57.6)
Dose, median (range), mg/kg	18.6 (11.3–32.2)	15.0 (12.2–22.2)	13.3 (3.4–33.3)	13.6 (3.4–33.3)
Participants with any anemia, n (%)	0 (0)	19 (76.0)	33 (39.3)	52 (46.4)
Anemia events, n	0	50	96	146
Grade 1 (mild)	0	14	48	62
Grade 2 (moderate)	0	14	25	39
Grade 3 (severe)	0	18	11	29
Grade 4 (potentially life-threatening)	0	4	12	16

Abbreviations: AUC, area under the concentration-time curve; WAZ, Weight-for-age z.

Separate parameter estimates of clearance, volume of distribution, and absorption rate constant were sequentially assessed for individuals with a WAZ score less than −2. Inclusion of WAZ scores did not result in improvement of the model fit and was therefore not retained in the final model. Estimation of between-subject variability on the absorption rate constant significantly improved the model (difference in Objective Function Value [dOFV], –13.57; *P* < .01). Additionally, covariance in between-subject variability estimates of clearance and volume of distribution further improved the model (dOFV, −17.32; *P* < .01). Overall, final model parameter estimates were like the initially described population pharmacokinetic model and are summarized in Table 2. A visual predictive check of the final model is included in Supplementary Figure 1. The linezolid AUC_0–24_ average in the study participants compared with the simulated linezolid AUC_0–24_ using the updated model with doses according to current linezolid dosing recommendations is included in Supplementary Figure 2.

**Table 2.** Final Model Parameter Estimates

Parameter	Original Pharmacokinetic Data Estimate (RSE)	Expanded Pharmacokinetic Data^a^ Estimate (RSE)
Ka (h^−1^)	0.77 (25)	0.982 (9.7)
CL/F (L/h/kg)	4.73 (8)	4.78 (9.6)
V/F (L)	54.8 (8)	58.1 (5.2)
BSV_CL/F_ (%)—shrinkage %	37 (16)	55.2 (32.1)—14.5
BSV_V/F_ (%)—shrinkage %	32 (23)	18.9 (38.1)—20.2
BSV_Ka_ (%)—shrinkage %	…	79.4 (23.3)—34.6
Correlation CL-V (%)	…	10
Additive error (mg/L)	0.78 (91)	1.83 (14.8)
Proportional error (%)	25 (30)	20.7 (31.4)

Abbreviations: BSV, between-subject variability; CL, clearance; F, bioavailability; Ka, absorption rate constant; RSE, relative standard error; V, volume of distribution.

^a^Expanded pharmacokinetic data refers to the original data plus the additional new pharmacokinetic data.

### Anemia

**Figure 2.**
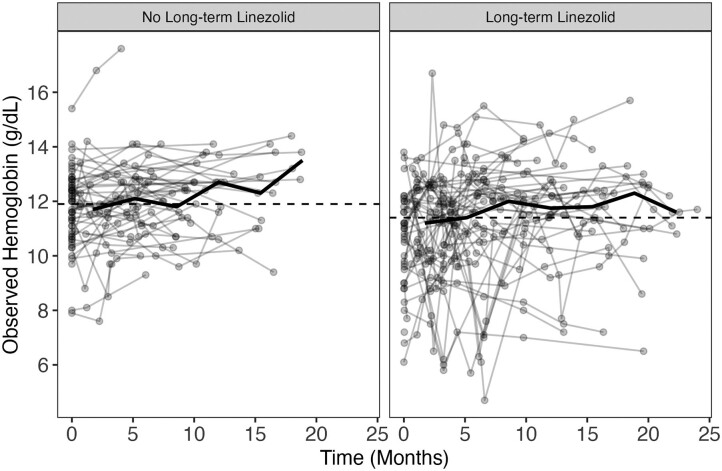
Hemoglobin concentrations over time grouped by linezolid exposure group. The dashed lines represent overall median hemoglobin, and the solid line represents the change in median over time for each exposure group. Overall median for “no long-term linezolid” and “long-term linezolid” were 11.8 g/dL and 11.4 g/dL, respectively.

**Figure 3.**
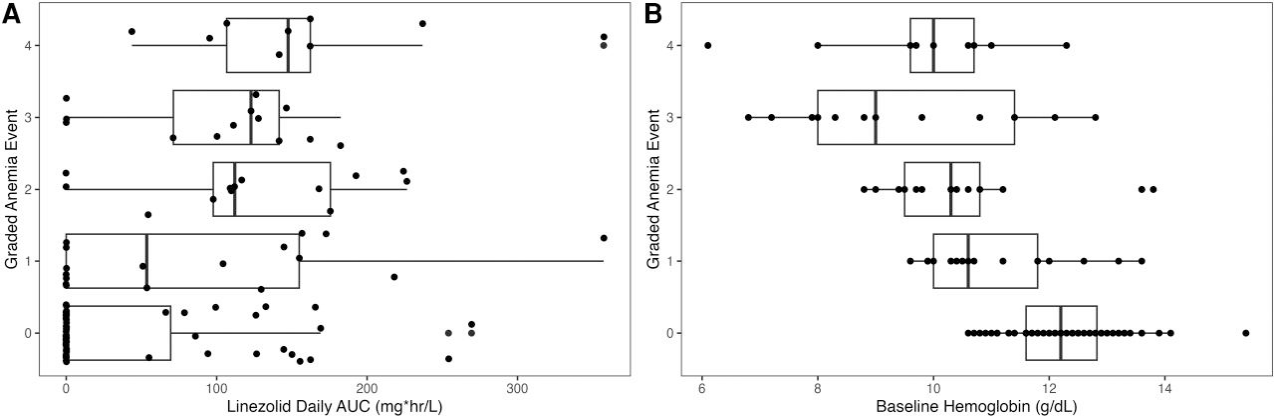
Most severe anemia event experienced subject by grade versus linezolid AUC_0–24_ per participant (*A*) and baseline hemoglobin (*B*). Abbreviation: AUC, area under the concentration-time curve.

Changes in hemoglobin over time among participants are depicted in Figure 2. The graded incidence of anemia compared with baseline hemoglobin and linezolid AUC_0–24_ is shown in Figure 3. Anemia events are summarized in Supplementary Tables 2 and 3. Forty-two patients did not have a true baseline hemoglobin measured. In these children, the first recorded hemoglobin was a median of 41 days into treatment (standard deviation, ± 28 days). These individuals were included in the safety analysis. A total of 52 participants (46.4%) experienced at least 1 anemia event. When the full dataset was evaluated, long-term linezolid increased the probability of experiencing both an anemia event of any grade (odds ratio [OR], 9.71; 95% confidence interval [CI], 4.0–23.5; *P* < .001) and a grade 3 or 4 anemia event (OR, 4.43; 95% CI, 1.2–16; *P* = .02).

The final model in which statistical significance of each independent variable was assessed using a Wald test included linezolid AUC_0–24_ (*P* = .0136) and baseline hemoglobin (*P* < .001; Table 3), where patients with no long-term linezolid were assigned an AUC_0–24_ of 0 mg/h × L to include this long-term effect variable without diminishing the AUC_0–24_ impact. While the number of children who received doses above the current linezolid dosing recommendations was significant in the final model, it was omitted, given its high correlation with linezolid AUC_0–24_. ORs and their CIs of univariable and the final multivariable models are included in Table 3.

**Table 3.** Odds Ratios for Ordinal Logistic Regression Model for Predicting Graded Anemia Event as Determined by Division of AIDS Criteria

	Univariable			Multivariable		
Characteristic	OR	95% CI	AIC	OR	95% CI	AIC
Baseline hemoglobin	0.399	.296–.522	253.6	0.379	.276–.505	233.5
Linezolid area under the time-concentration curve over 24 hours at steady state	1.011	1.006–1.016	262.2	1.012	1.007–1.017	
Duration of linezolid	1.002	1.001–1.003	293.4			
Female sex	1.650	.813–3.392	304.4			
Age	1.092	1.023–1.169	299.3			
Weight-for-age *z* score	0.811	.526–1.212	308.3			
Dose above current recommendations^a^	5.429	2.223–13.632	292.6			

Abbreviations: AIC, Akaike’s information criterion; CI, confidence interval; OR, odds ratio.

The empty cells under the multivariable section are for those variables not included in the final multivariable model.

^a^Given dose recommendations changed after the development of the initial population pharmacokinetic model. Some of the data that were collected prior to current recommendations contained doses above what is currently recommended and were evaluated in the ordinal logistic regression model.

**Figure 4.**
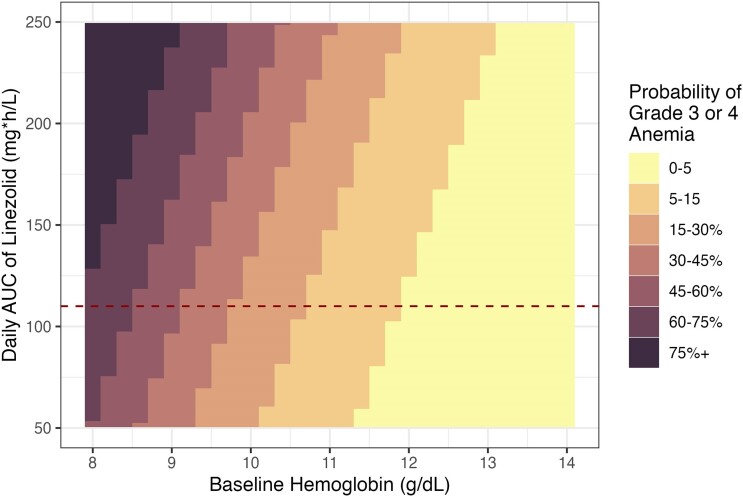
Probability of experiencing a grade 3 or 4 anemia event generated from the regression model given baseline hemoglobin and when starting long-term linezolid treatment for tuberculosis and average daily exposure of linezolid. The dashed line corresponds to the target AUC of 107 mg × h/L. Abbreviation: AUC, area under the concentration-time curve.

**Figure 5.**
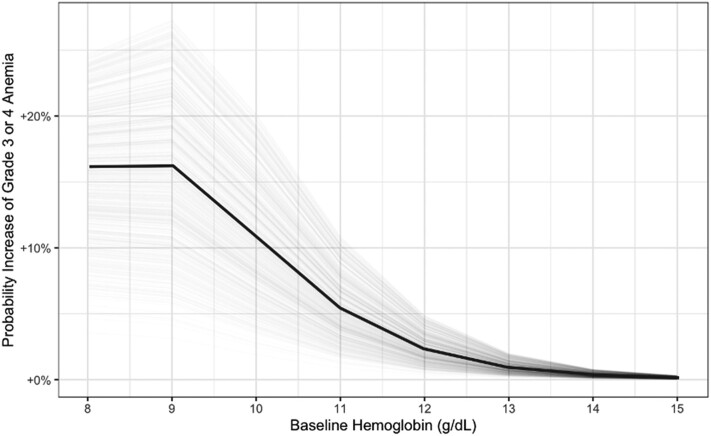
Increase in probability of a grade 3 or 4 anemia event based on the ordinal logistic regression model when starting long-term linezolid for tuberculosis with the recommended dose and formulation [9] at a patient's given baseline hemoglobin.

The probability of experiencing grade 3 or 4 anemia, as predicted by the ordinal regression model given baseline hemoglobin and linezolid AUC_0–24_, is shown in Figure 4. Since the average daily linezolid AUC_0–24_ is not normally available as a metric to clinicians who routinely use linezolid for the treatment of RR/MDR-TB in the field, we ran simulations of the predicted linezolid AUC_0–24_ for those who were receiving the currently recommended dose of linezolid and reported their increase in the probability of developing grade 3 or 4 anemia given a baseline hemoglobin measurement, which would be more clinically informative (Figure 5). The probability of each anemia event versus linezolid AUC_0–24_ and baseline hemoglobin values are presented in Supplementary Figures 2 and 3. The probability of developing grade 3 or 4 anemia compared with baseline hemoglobin by linezolid exposure is presented in Supplementary Figures 4 and 5.

## DISCUSSION

The prospective linezolid pharmacokinetic data evaluated in this study validate the previously described population pharmacokinetic model of linezolid in children. Since no changes were necessary to improve the model fit with this additional data, no changes are needed in the current dosing algorithm for children and adolescents to achieve exposures that are comparable to those of adults who receive a standard 600-mg daily linezolid dose [7].

In developing the model for predicting linezolid-related anemia and determining the risk of experiencing anemia and higher-grade anemia, baseline hemoglobin and the average daily linezolid exposure were the 2 most important factors. In the evaluation of linezolid-related adverse effects in adults treated for MDR-TB, there have been mixed results in identifying the relationship between adverse effects and linezolid trough concentrations, with 2 studies not finding an association [15, 16] and 1 study finding a significant relationship between trough linezolid concentrations and toxicity due to a drop in platelets, but not for hemoglobin [6]. Additionally, there are multiple drivers of anemia, including poor nutrition, helminth infection, and iron deficiency. The WHO estimates the prevalence of anemia among children aged 6–59 months in South Africa and India to be 44.4% and 53.4%, respectively [17]. This background epidemiological and specific finding helps to contextualize our finding that baseline hemoglobin is the most important predictor of experiencing anemia while receiving long-term linezolid for RR/MDR-TB. However, high exposure to linezolid, especially if receiving doses higher than currently recommended for a child's given weight, added to the risk of developing anemia. Additionally, while the duration of linezolid treatment was significant in univariable model analysis, greater granularity of treatment durations is needed to characterize this effect more accurately. In our study, this effect of treatment duration was indistinguishable from including a dichotomous category of “long-term” linezolid versus “no long-term” linezolid treatment. Notably, the linezolid doses that were received were in line with the dosing recommendations at the time of treatment in the various cohorts, with lower doses used following initial analysis in the MDRPK1 study.

In children with baseline hemoglobin at or above the normal value (≥11 g/dL), starting linezolid was still associated with a meaningful risk (0%–15%) of developing linezolid-induced grade 3 or 4 anemia. However, children who started linezolid with even moderately low baseline hemoglobin (10 g/dL) also had substantial risk of developing grade 3 or 4 anemia (15%–45%). This highlights the importance of baseline hemoglobin assessment at the onset of RR/MDR-TB treatment regimens that include linezolid and the need for frequent monitoring of hemoglobin levels in all children who require linezolid. In children, monitoring linezolid-related anemia every 2 weeks for the first month of treatment and then monthly thereafter should be considered and repeated earlier if a concerning decrease in hemoglobin (or other toxicities) is present [6]. Several crucial issues and practical questions remain, including acknowledging that more frequent hemoglobin monitoring is often difficult in routine programmatic settings. Additional studies are needed to address the balance between preventing anemia toxicity and maintaining treatment efficacy with linezolid. Evaluation of the use of safer and more effective oxazolidines is an important priority for children and adolescents.

This study helps to clarify the added risk of anemia when starting linezolid in children (Figure 5, Supplementary Figure 5). Given that the risk of developing anemia during RR/MDR-TB treatment that includes linezolid is associated with linezolid exposures, selection of appropriate doses and formulation of linezolid for the weight and formulation tolerability of each child are extremely important. Newly developed, dispersible, functionally scored, pediatric linezolid formulations may facilitate more accurate dosing compared with the traditional suspension used for young children. We explored a dichotomous variable that indicated if participants received doses of linezolid above the currently recommended weight-based dosing, which was found to be a significant predictor of anemia (Supplementary Figure 3). Of the participants who contributed pharmacokinetic data while receiving the currently recommended lower linezolid doses, 9 (38%) had hemoglobin levels that corresponded to grade 1 or 2 anemia and only 1 (4%) had a hemoglobin level that corresponded to grade 3 or 4 anemia.

We did not include analysis of the relationship between linezolid pharmacokinetics and treatment outcome. Current linezolid dosing recommendations, which are based on extrapolation from adult pharmacokinetic–pharmacodynamic data, suggest an optimal linezolid oral dose of 600 mg daily for adults. Additional work to explore the relationship between linezolid pharmacokinetics and efficacy is needed to evaluate current linezolid dosing recommendations across the pediatric age range and optimize treatment efficacy while minimizing the risk of anemia and other linezolid-related adverse effects.

There are several limitations to this study. Data were from only 2 countries, South Africa and India. Also, only 2 children (1.75%) were diagnosed with human immunodeficiency virus (HIV), and the potential effects of HIV on linezolid pharmacokinetics and risk of anemia are therefore not clear. Another limitation is that not all baseline hemoglobin data were “true” pre-linezolid initiation, given the timing of enrollment. Also, anemia events after discontinuation of linezolid were not included. Additionally, full blood counts and assessment of peripheral neuropathy and optic neuritis were not available. Because this study included only 2 patients aged <6 months, extrapolation to infants and neonates is limited. More research is needed on the subtypes of anemia as well as thrombocytopenia, neutropenia, and neuropathy, all of which are significant concerns in people who receive long-term linezolid treatment. Additionally, the population pharmacokinetic model that was developed, which was supported by greater sample sizes and larger pools of covariates to explore, will likely further improve the model.

In conclusion, these additional linezolid pharmacokinetic data validate the previously developed population pharmacokinetic model and confirm that currently recommended doses for children and adolescents are appropriate for achieving linezolid exposures that are comparable to those of adults who receive a dose of 600 mg daily [11]. Anemia, including grade 3 and 4 events, was frequent in children. While baseline hemoglobin was the main driver of developing anemia, the use of linezolid for RR/MDR-TB treatment further increases the risk of developing high-grade anemia. Baseline and frequent hematological safety monitoring is therefore essential to ensure both treatment efficacy and reduce the risk of anemia.

## Supplementary Data

Supplementary materials are available at *Clinical Infectious Diseases* online. Consisting of data provided by the authors to benefit the reader, the posted materials are not copyedited and are the sole responsibility of the authors, so questions or comments should be addressed to the corresponding author.

## Supplementary Material

ciae497_Supplementary_Data

## Notes

***Author contributions*.** A. B., B. P. S., H. R. D., A. J. G.-P., and R. M. S. wrote the protocol and conceptualized the study. J. F., S. S., H. R. D., H. S. S., L. L., A. C. H., and A. J. G.-P. screened the studies and extracted the data. J. T. B., A. B., and B. P. S. performed the statistical analysis, wrote the first draft of the manuscript with input from R. S., J. B., A. B., and B. P. S., and accessed and verified the data. All authors reviewed the manuscript and approved the final version, had full access to all the data collected in the systematic review, and had responsibility for the decision to submit for publication.

***Acknowledgments*.** The authors thank all the children, families, caregivers, and healthcare workers who participated in the studies included in this analysis.

***Financial support*.** This work was supported by Unitaid through the BENEFIT Kids project. Unitaid accelerates access to innovative health products and lays the foundations for their scale-up by countries and partners. This work was also supported by the Stop TB Partnership's Global Drug Facility to support the roll-out of child-friendly formulations of second-line drugs.
