# Supplementary material for: Linezolid Pharmacokinetic-Anemia Modeling in Children With Rifampicin-Resistant Tuberculosis
Source: Clin Infect Dis. 2024 Oct 18;79(6):1495–502. doi: 10.1093/cid/ciae497 (PMC11650862; doi:10.1093/cid/ciae497)
Supplement: ciae497_Supplementary_Data [file ciae497_supplementary_data.docx]

Linezolid Pharmacokinetic-Anemia Modeling in Children with Rifampicin-Resistant Tuberculosis

**SUPPLEMENTARY MATERIAL**

**Supplemental Table 1. Summary of New Participants’ Linezolid Pharmacokinetic Data**

|  | |
| --- | --- |
| Participants – n | 24 |
| Sex  F – n (%)  M – n (%) | 10 (41.7)  14 (58.3) |
| Dose – median (range) in mg/kg | 11.4 (4.7 – 18.9) |
| Weight – median (range) in kg | 13.0 (6.3 – 52.5) |
| Age – median (range) in years | 4.5 (0.6 – 12.1) |
| Age > 10 years – n (%) | 5 (20.8) |
| HIV-positive – n (%) | 2 (8.3) |
| Linezolid Doses – median (range) in mg  Forms – n (%)  Whole Tablet or Capsule  Crush Tablet or Opened Capsule  Suspension | 300 (50 – 600)  14 (58.3)  4 (16.7)  6 (25.0) |
| Linezolid Exposure Groups – n  Single Dose – PK Study  Multiple Doses – Treatment | 10  14 |
| Linezolid Concentrations – n  Total number of concentrations - n  BLQ – n (%)  Concentration – median (range) in mg/L  Sampling Occasion – n participants (combined total number of concentrations per occasion)  Occasion 1  Occasion 2  Occasion 3 | 63  3 (4.8%)  8.34 (0.05 – 28.8)  19 (48)  3 (7)  2 (8) |

| **Supplemental Table 2. Anemia Severity** | |
| --- | --- |
| ***All Anemia Events*** | |
| Total Number of Any Anemia Event – n  No Long-term Linezolid  Long-term Linezolid | 30  116 |
| Severity of Anemia Events in No Long-term Linezolid – n (%)  Grade 1 (Mild)  Grade 2 (Moderate)  Grade 3 (Severe)  Grade 4 (Potentially life-threatening) | 19 (63.3)  6 (20.0)  5 (16.7)  0 (0) |
| Severity of Anemia Events in Long-term Linezolid – n (%)  Grade 1 (Mild)  Grade 2 (Moderate)  Grade 3 (Severe)  Grade 4 (Potentially life-threatening) | 43 (29.3)  33 (22.4)  24 (16.3)  16 (11.6) |
| ***Single Most Severe Anemia Event*** |  |
| Severity of Most Severe Anemia in No Long-term Linezolid – n (%)  None  Mild  Moderate  Severe  Potentially Life-Threatening | 43 (82.7)  7 (13.5)  1 (1.9)  1 (1.9)  0 (0) |
| Severity of Most Severe Anemia in Long-term Linezolid – n (%)  None  Mild  Moderate  Severe  Potentially Life-Threatening | 18 (30.0)  13 (21.7)  10 (16.7)  10 (16.7)  9 (15.0) |

| **Supplemental Table 3. Graded Anemia by Age and Daily Dose for Individuals on Long-term Linezolid** | | | | | |
| --- | --- | --- | --- | --- | --- |
| ***All Anemia Events*** | | | | | |
| *Age Group* | *Dose Group* | *Anemia Grade* | | | |
|  |  | 1 - Mild | 2 - Moderate | 3 - Severe | 4 - Potentially Life-Threatening |
|  | <10 mg/kg | 13 | 10 | 8 | 6 |
| < 10 years | 10 - 20 mg/kg | 14 | 7 | 6 | 4 |
|  | >20 mg/kg | 2 | 1 | 1 | 0 |
|  | <10 mg/kg | 6 | 7 | 4 | 4 |
| ≥ 10 years | 10 - 20 mg/kg | 8 | 8 | 5 | 2 |
|  | >20 mg/kg | 0 | 0 | 0 | 0 |


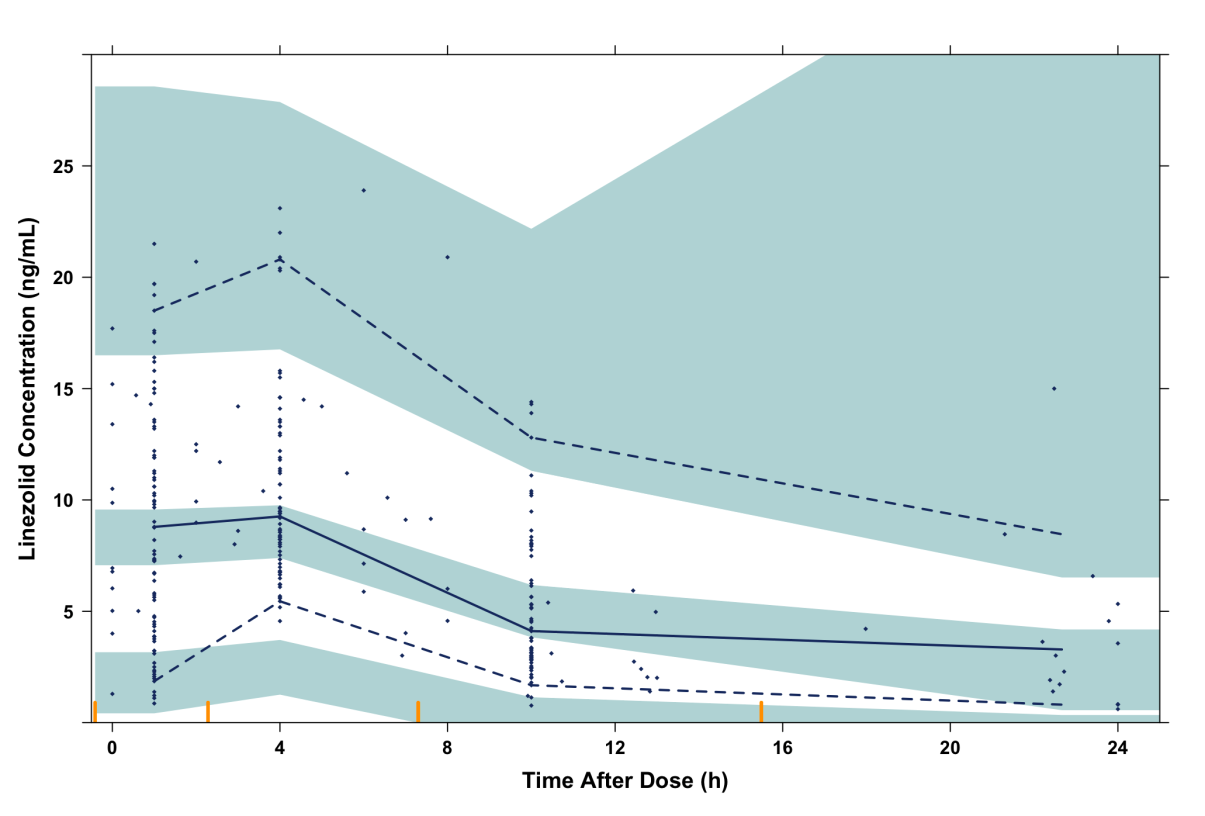


**Supplemental Figure 1.** Visual predictive check of the final linezolid population pharmacokinetic model where the dots represent the raw observations, the solid line is the median of the observed data, the dotted lines are the 5th and 95th percentile of the observed data, and the shaded areas represent the prediction interval of the 5th, median, and 95th percentiles of the model predictions, and the yellow x-axis ticks corresponded to the binning of the data.


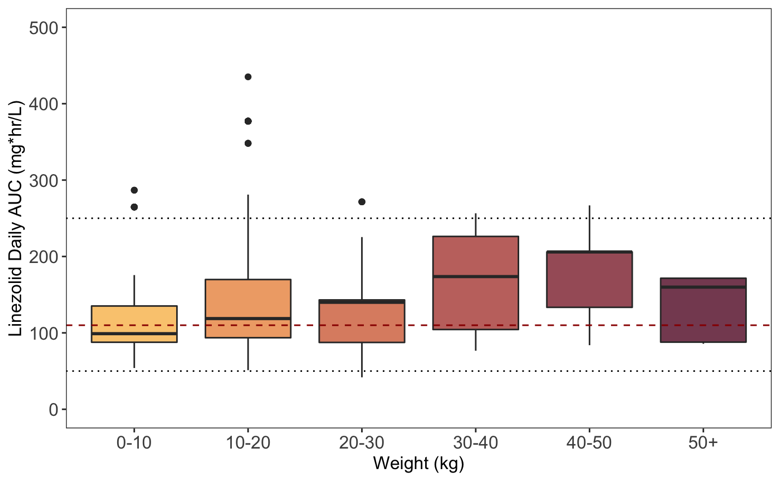

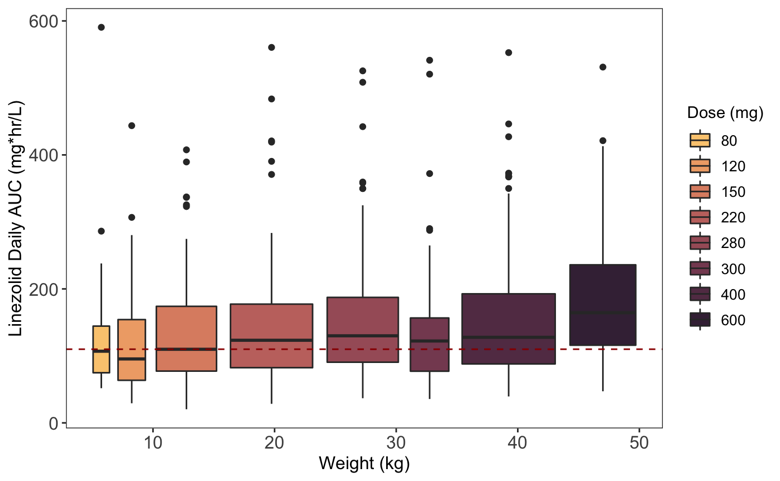


(A)

(B)

**Supplemental Figure 2.** (A) linezolid AUC_0-24_ as determined by the updated population pharmacokinetic model for participants by weight-band who received long-term linezolid included both the original linezolid PK data participants and the new linezolid PK data participants. (B) Simulation of exposures with current linezolid dosing guidelines utilizing the updated linezolid population pharmacokinetic model. The width of the boxplots corresponds to the weight-band across each group.


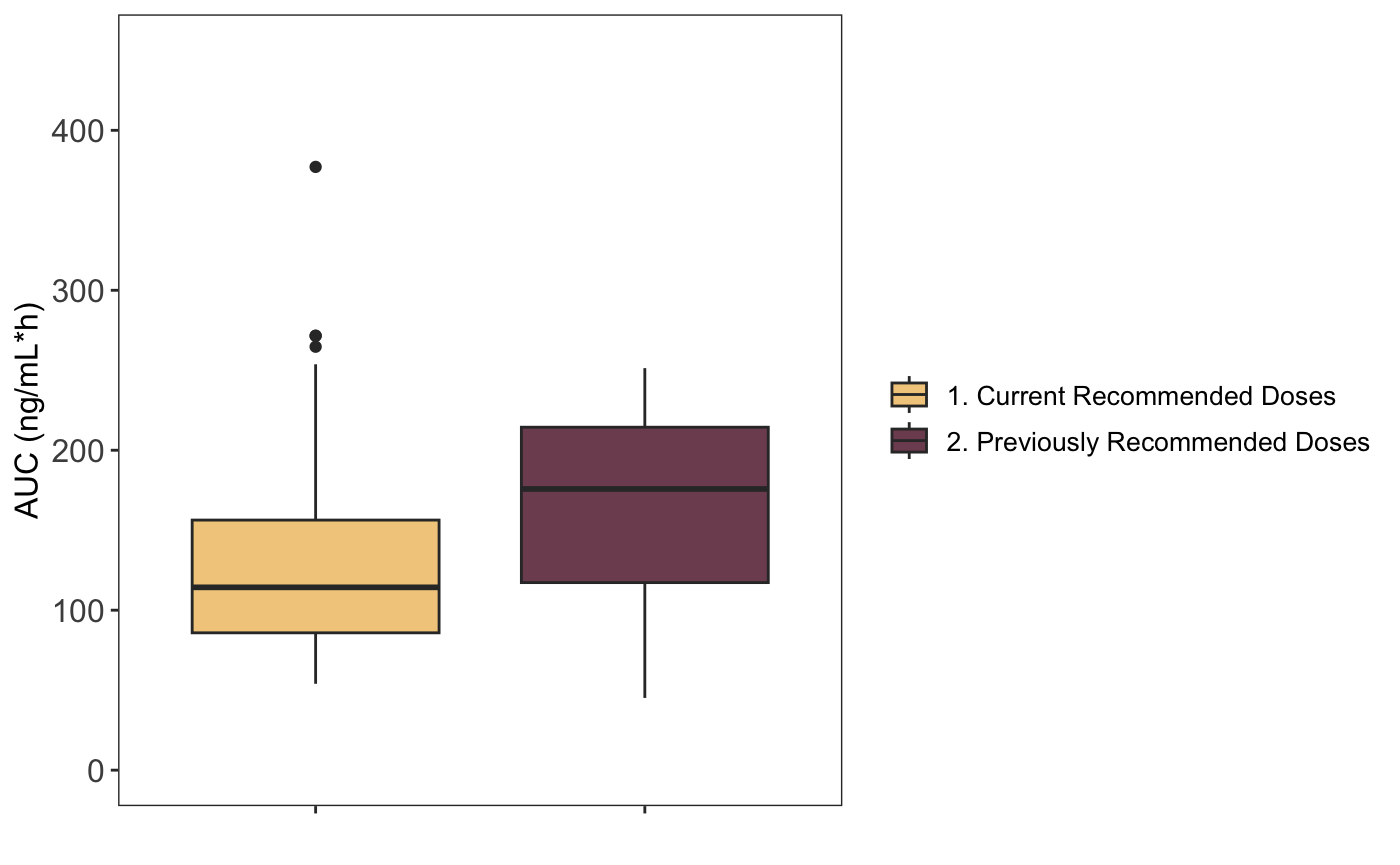


**Supplemental Figure 3.** Linezolid AUC_0-24_ as determined by the updated population pharmacokinetic model for participants grouped by if any doses they received were at or above the recommended weight-based dose.


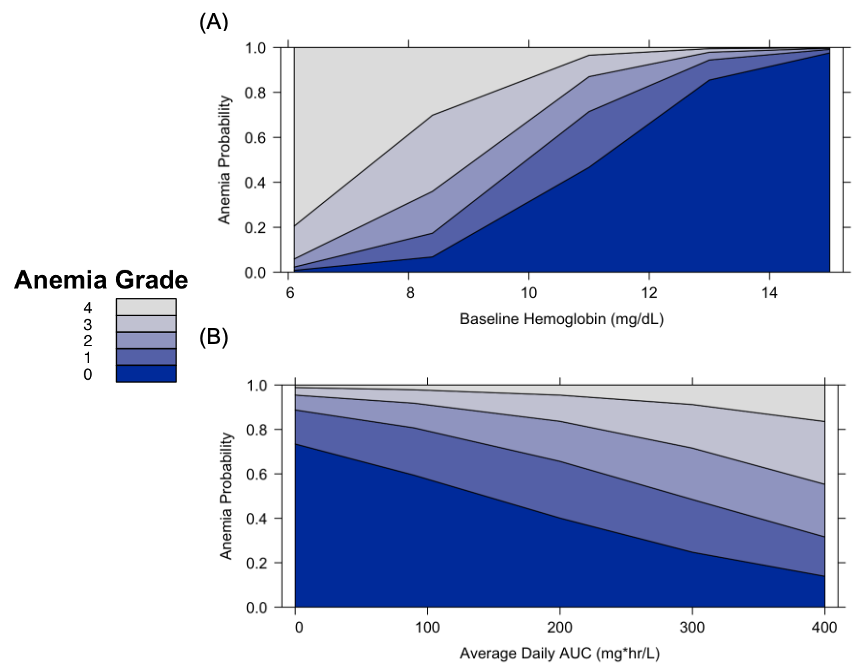


**Supplemental Figure 4.** Probability of a child experiencing any event of anemia while on long-term linezolid for TB treatment by anemia grade based on (A) baseline hemoglobin and (2) average daily AUC of linezolid.

**
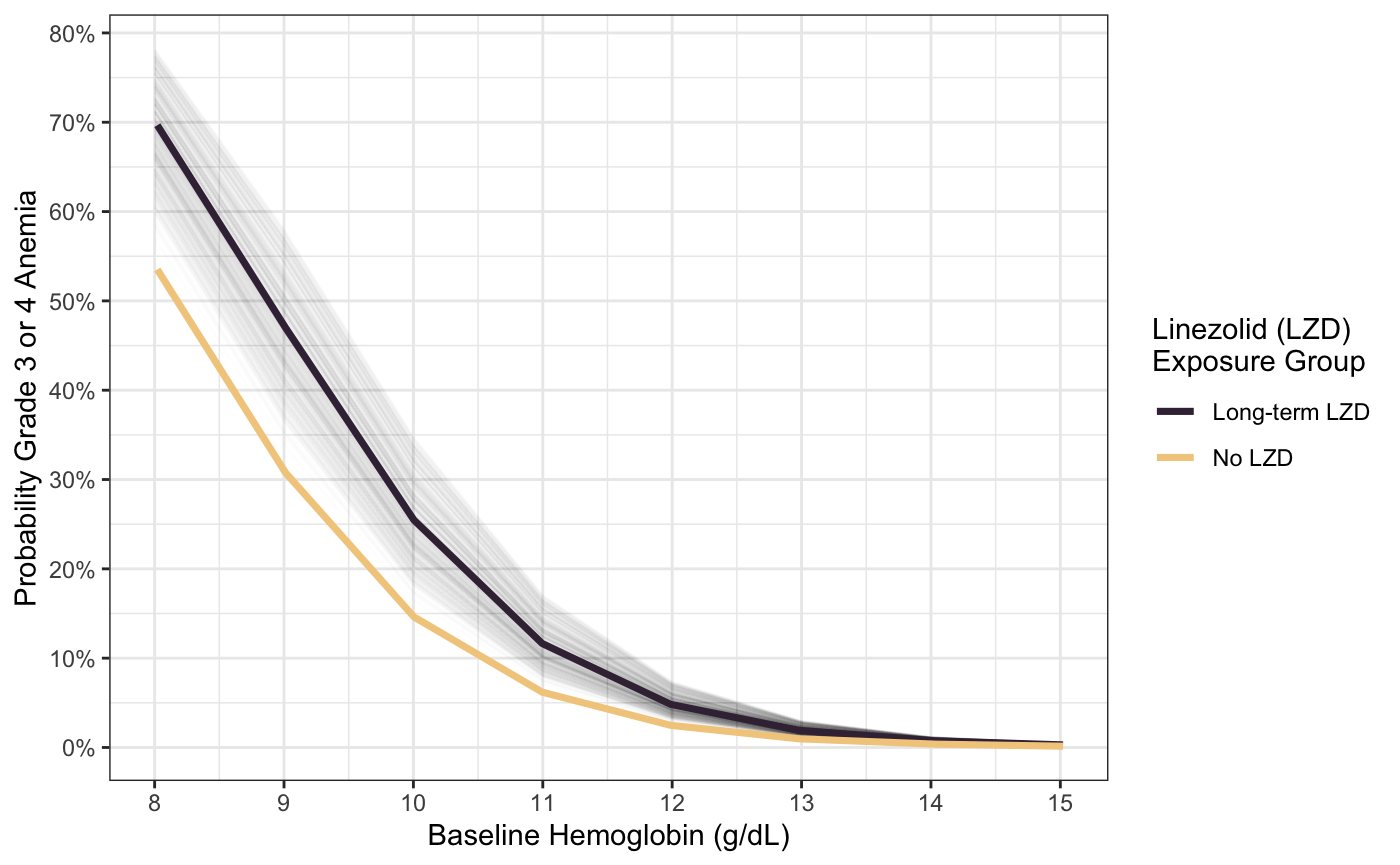
**

**Supplemental Figure 5.** Probability of a grade 3 or 4 anemia event when starting long-term linezolid for TB with the recommended dose and formulation^9^at a patient’s given baseline hemoglobin (black) and no linezolid.

**Supplemental Equation 1: Ordinal Logistic Regression**

logit(P(0>Y<1)) = -9.9834 (- 0.96964* BASELINEHEMOGLOBIN) (+0.01152*AVGDAILYAUC)

logit(P(1>Y<2)) = -8.7808 (- 0.96964* BASELINEHEMOGLOBIN) (+0.01152*AVGDAILYAUC)

logit(P(2>Y<3)) = -7.6916 (- 0.96964* BASELINEHEMOGLOBIN) (+0.01152*AVGDAILYAUC)

logit(P(3>Y<4)) = -6.1892 (- 0.96964* BASELINEHEMOGLOBIN) (+0.01152*AVGDAILYAUC)

**Supplementary Material. Nonmem code**

$PROBLEM dummy

$INPUT dummy

$DATA dummy

$SUBROUTINE ADVAN6 TOL=4

$MODEL NCOMP=3 COMP=(DEPOT DEFDOS) COMP=(CENTRAL DEFOBS)

COMP=(AUC)

$ABBREVIATED COMRES=2

$PK

;;;;;;;;;;;; PK MODEL ;;;;;;;;;;;;;;

; Allometric scaling definition

ALLOCL = (WT/70)**0.75

ALLOWT = (WT/70)

; Typical parameters definition

TVCL = THETA(1)

TVV = THETA(2)

TVF1 = 1

; Parameters definition

F1 = TVF1

CL = TVCL*ALLOCL*EXP(ETA(1)) ; CL, L/h [systemic clearance]

V = TVV*ALLOV*EXP(ETA(2)) ; V, L [central volume]

K20 = CL/V

TVKA= K20+THETA(3)

KA = TVKA*EXP(ETA(3))

S2 = V ; Scalar

AUCINF = F1*AMT/CL

IF(NEWIND.LE.1) THEN ; Assign negative Cmax Tmax for the new subject

COM(1)=-1 ; Holder of Cmax

COM(2)=-1 ; Holder of Tmax

ENDIF

$DES

DADT(1) = -KA*A(1) ; Absorption

DADT(2) = KA*A(1)-K20*A(2) ; Central

DADT(3) = A(2)/V ; AUC

CP=A(2)/V

CT=A(2)/S2 ;for simulation

IF(CT.GT.COM(1)) THEN

COM(1)=CT

COM(2)=T

ENDIF

REP=IREP

$ERROR

AUC=A(3)/S2

PKS=A(2)/V

IPRED = F

PROP=THETA(4)

ADD=THETA(5)

W=SQRT(PROP**2*IPRED**2+ADD**2)

IRES = DV - IPRED

IWRES = IRES/W

Y = IPRED + W*EPS(1)

CMAX = COM(1)

TMAX = COM(2)

$THETA (0,4.78254,25) FIX ; CL,L/h [systemic clearance]

(0,58.1292,100) FIX ; Vc,L [central volume]

(0,0.982,10) ; KA

(0,0.20748,10) ; 4 proprotional

(0,1.83731,50) ; 5 additive

$OMEGA BLOCK(2)

0.305884 ; CL IIV

0.104564 0.01413721

$OMEGA 0.630436 ; KA IIV

$SIGMA 1 FIX

$ESTIMATION MAXEVAL=9999 METHOD=1 PRINT=5 SIG=3 NOABORT ;FOCEI

;$SIM ONLYSIM(123456) SUBPROBLEMS=1

$COVARIANCE PRINT=E

$TABLE dummy
